# Aurora-A/FOXO3A/SKP2 axis promotes tumor progression in clear cell renal cell carcinoma and dual-targeting Aurora-A/SKP2 shows synthetic lethality

**DOI:** 10.1038/s41419-022-04973-9

**Published:** 2022-07-13

**Authors:** Pu Li, Tingting Chen, Peng Kuang, Fujun Liu, Zhongmin Li, Fangfang Liu, Yu Wang, Wenfeng Zhang, Xiuyu Cai

**Affiliations:** 1 State Drug Clinical Trial Agency, The First Affiliated Hospital of Nanchang University, Nanchang, 330052 China; 2 Department of Urology, The Second Affiliated Hospital of Third Military Medical University (Army Medical University), Chongqing, 400037 China; 3 Department of Oncology, The First Affiliated Hospital of Nanchang University, Nanchang, 330052 China; 4 Department of Obstetrics and Gynecology, The First Affiliated Hospital of Nanchang University, Nanchang, 330006 China; 5 Department of Infectious Disease, The First Affiliated Hospital of Nanchang University, Nanchang, 330052 China; 6 Department of Nephrology, The First Affiliated Hospital of Nanchang University, Nanchang, 330052 China; 7 Department of VIP Inpatient, State Key Laboratory of Oncology in South China, Cancer Center, Sun Yat-sen University, Guangzhou, 510060 China

**Keywords:** Targeted therapies, Renal cell carcinoma

## Abstract

Renal cell carcinoma (RCC) is a common malignant tumor in the world. Histologically, most of RCC is classified as clear cell renal cell carcinoma (ccRCC), which is the most prevalent subtype. The overall survival of patients with ccRCC is poor, thus it is urgent to further explore its mechanism and target. S-phase kinase-associated protein 2 (SKP2) is overexpressed in a variety of human cancers and is associated with poor prognosis by enhancing tumor progression. However, it is unclear whether or how SKP2 is involved in ccRCC progression. Here, we reported that overexpression of SKP2 enhanced cell proliferation of ccRCC, while SKP2 depletion exhibited the opposite effect. Bioinformatic analyses found that SKP2 was positively correlated with Aurora-A (Aur-A) in ccRCC. The protein and mRNA levels of SKP2 were elevated or reduced by Aur-A overexpression or silencing, respectively. It was further found that Aur-A caused an increase phosphorylation of FOXO3A, which is a negatively transcription factor for SKP2. Interestingly, SKP2 mediated ubiquitylation and degradation of FOXO3A depend on the kinase activity of Aur-A. The combination of Aur-A inhibitor MLN8237 and SKP2 inhibitor SZL P1-41 showed a synergistic tumor growth inhibition in vivo and in vitro of ccRCC models. Thus, our data reveal that Aurora-A/FOXO3A/SKP2 axis promotes tumor progression in ccRCC, and the double inhibition of SKP2 and Aur-A shows significant synergistic effect, which indicates a potential new therapeutic strategy for ccRCC.

## Introduction

Kidney cancer accounts for 3.7% of all new cancers, with an annual global incidence of almost 300,000 cases leading to 111,000 deaths [1]. The disease encompasses more than ten histological and molecular subtypes, of which clear cell renal cell carcinoma (ccRCC) is the major histologic subtype [2]. Immunotherapeutic drugs, such as interferon-α and interleukin-2 have been the primary treatment options, despite the low response rate and high toxicity in the past decades [3, 4]. Despite the use of novel therapeutic approaches involving vascular endothelial growth factor, platelet derived growth factor and PI3K, the response rate of these drugs is not high [5]. Thus, insight into the molecular mechanisms driving tumor progression of ccRCC is urgently required to support the development of more effective therapeutic strategies in the future.

S-phase kinase-associated protein 2 (SKP2), also known as P45 or FBXL1, is a member of the F-box protein family and is involved in ubiquitination, cell cycle control and signal transduction in the form of the Skp2-SCF complex (Cul1-Rbx1-SKP1-F-box^SKP2^) [6, 7]. SKP2 acts as a substrate recognition factor in SCF complex [8]. SKP2 has been proved to be an oncogene [9], which is overexpressed in prostate cancer [10], melanoma [11], nasopharyngeal carcinoma [12], and breast cancer [13]. A clinical retrospective study showed that preoperative enhanced expression of SKP2 could predict 94% of breast cancer patients to develop resistance to cyclophosphamide/doxorubicin/5-fluorouracil [14]. On the one hand, knockout of SKP2 significantly increased the sensitivity of Her2 positive breast cancer cells to Herceptin [15], on the other hand, SKP2 silencing or inactivation can restore non-small cell lung cancer sensitivity to gefitinib treatment [16]. However, the biological functions of SKP2 in ccRCC are still unknown. Therefore, in-depth study of the molecular mechanism of Skp2 expression regulation in ccRCC cells is of great clinical significance for a comprehensive understanding of the drug resistance mechanism of ccRCC and looking for new targeted treatment strategies.

Many protein kinases are key regulators of cell cycle progression and are frequently deregulated in cancer [17, 18]. Among them, Aurora kinases have attracted much interest as promising targets for cancer treatment [19]. Aurora-A (Aur-A), also known as STK15, BTAK, and Aurora-2, a member of the mitotic serine/threonine Aurora kinase family, is essential in accurate timing of mitosis and maintenance of bipolar spindles [20, 21]. Aur-A dysregulation and overexpression are frequently correlated with chromosomal instability and clinical aggressiveness in malignancies, which are overexpressed in many human tumors, including primary breast cancer, colorectal cancer, hepatic carcinomas and ovarian cancer [22, 23]. Several recent studies have shown that overexpression of Aur-A in cancer cells can upregulate oncogenic signaling pathways such as PI3K/AKT and β-catenin [24]. In addition, there is evidence that Aur-A can regulate p73, a member of the p53 family [25]. A number of small molecule drug inhibitors of Aurora kinases are currently under development or testing for the treatment of cancer. Alisertib (MLN8237) is an investigational small molecule inhibitor that selectively inhibits Aur-A and has been shown in non-clinical studies to thereby induce cell-cycle arrest, polyploidy, and mitotic catastrophe [26]. However, the single-agent treatment of Alisertib is still limited by the modest side effects. The main side effects include febrile neutropenia, stomatitis, gastrointestinal toxicity, hypertension, and fatigue [27]. Alisetib combined with other chemotherapeutic agents or small molecular inhibitors may decrease the toxicity caused by single-agent treatment and enhance its anticancer effects.

Here, we reported that SKP2 expression was prominently increased both in ccRCC tumor tissues and cells, and the high expression of SKP2 predicted a worse survival of ccRCC. Overexpression of SKP2 enhanced cell proliferation of ccRCC, whereas SKP2 depletion exhibited the opposite effect. It was further found that Aur-A was positively correlated with SKP2 in ccRCC and regulated protein and mRNA levels of SKP2. Aur-A also caused an increase phosphorylation of FOXO3A, which its ubiquitylation and degradation by SKP2 mediating depend on the kinase activity of Aur-A. Simultaneously targeting both SKP2 and Aur-A showed a synergistic tumor growth inhibition in vivo and in vitro of ccRCC models, suggesting a potential new therapeutic strategy for ccRCC.

## Results

### SKP2 is overexpressed in ccRCC tissues and cells, and enhanced SKP2 promotes cell growth

**Fig. 1:**
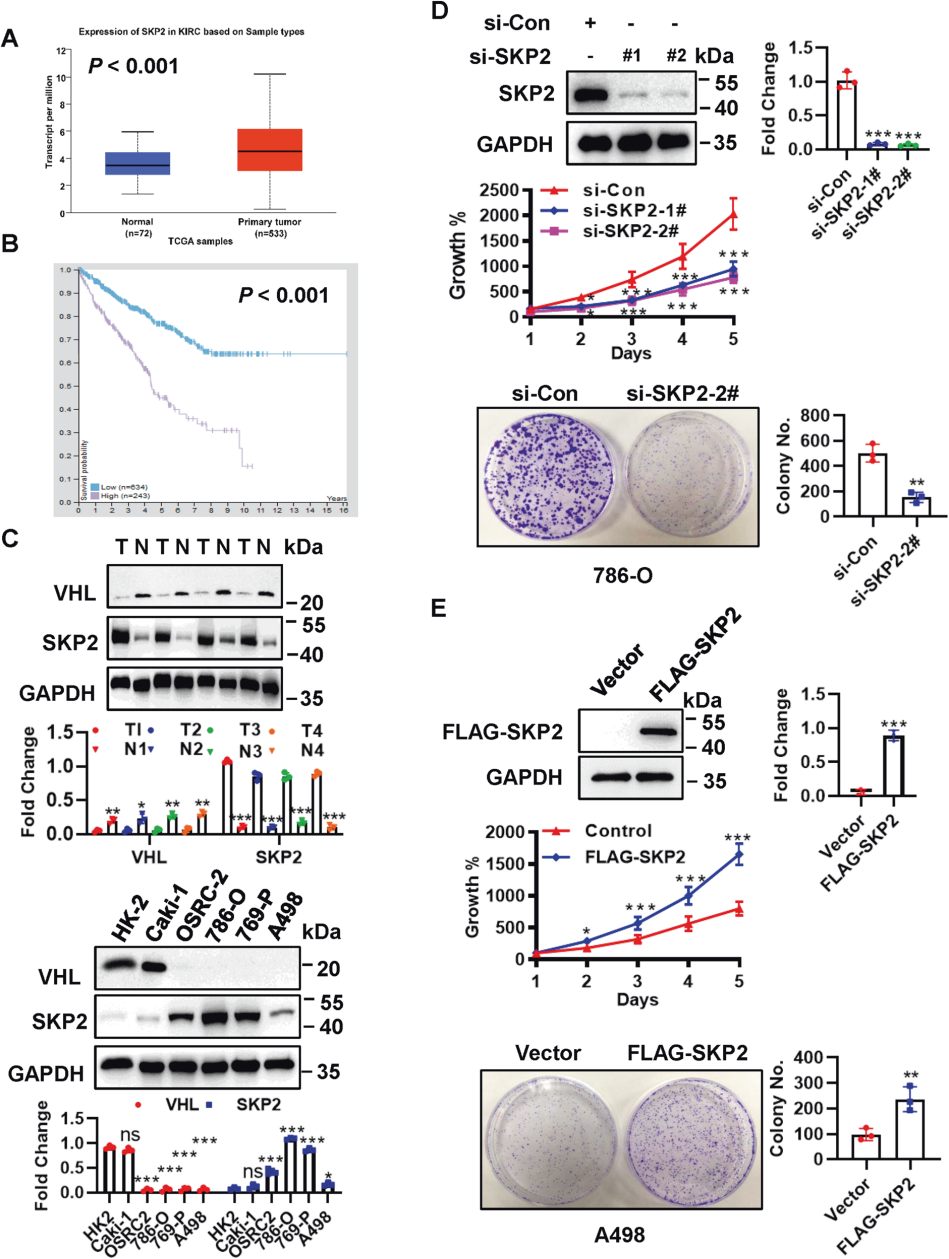
SKP2 is up-regulated both in ccRCC tissues and cells, and enhanced SKP2 can promote cell growth. **A** Bar graph showing expression values of SKP2 gene using the study dataset, and data from http://ualcan.path.uab.edu. The 95% confidence interval was also displayed. **B** Protein expression of SKP2 in ccRCC and relationship with patient survival: Kaplan–Meier survival analysis indicated that patient with higher expression of SKP2 was related to a worse overall survival (log-rank test, *p* < 0.001). **C** Immunoblotting (IB) analysis of SKP2 and VHL expression in ccRCC tissues (T) and normal adjacent tissues (N), and ccRCC cell lines. **D** Depletion SKP2 observably suppressed cell proliferation and clonogenic ability in ccRCC cells. 786-O cells were transfected siRNAs targeting SKP2, and followed by IB, CCK-8 and colony formation assay, respectively. **E** Overexpression SKP2 significantly stimulated cell proliferation and clonogenic ability in ccRCC cells. A498 cells were transfected with vector or FLAG-SKP2, and followed by IB, CCK-8 and colony formation assay, respectively. The protein levels were normalized to GAPDH. ns no significance; *p < 0.05, **p < 0.01, ***p < 0.001; *n* = 3 independent experiments; two-tailed paired or unpaired Student’s *t* test or one-way ANOVA with Dunnett’s test.

SKP2 is overexpressed in a variety of human cancers and associated with poor prognosis by enhancing tumor progression [7]. By bioinformatics analysis, we found that the overexpression of SKP2 in ccRCC was confirmed using The Cancer Genome Atlas (TCGA) database (Fig. 1A). Kaplan–Meier survival analysis indicated that patients with higher expression of SKP2 was related to a worse overall survival (Fig. 1B). We also collected four pairs of clinical samples (including tumor tissues and adjacent normal tissues) and five ccRCC cell lines to detect the protein level of SKP2, and further confirmed that SKP2 was highly expressed in ccRCC tumor tissues and cells (Fig. 1C). Next, we performed an siRNA-based knockdown experiment in 786-O ccRCC cells and found SKP2 depletion inhibited cell proliferation and clone-forming ability (Fig. 1D). Whereas overexpression of SKP2 significantly stimulated cell proliferation and clone-forming ability of A498 ccRCC cells (Fig. 1E).

### Aur-A is positively correlated with SKP2 and regulates SKP2 protein level upon its kinase activation in ccRCC

**Fig. 2:**
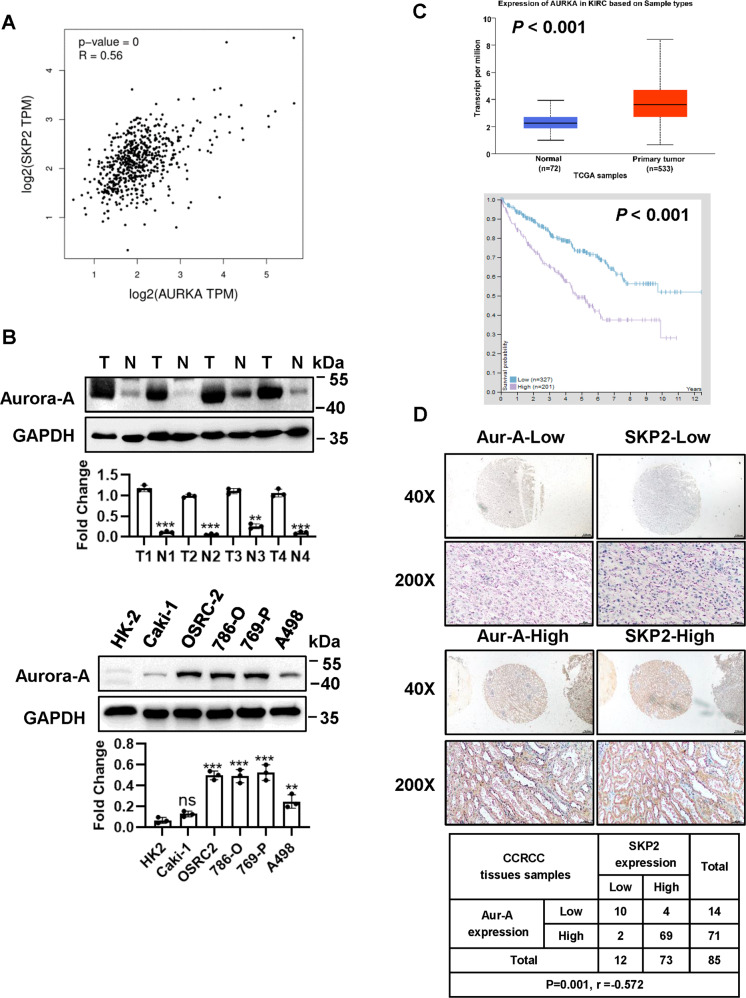
SKP2 is positively correlated with Aur-A. **A** SKP2 was extremely correlated positive correlation with Aur-A, and data from http://gepia.cancer-pku.cn/. **B** Basal level of Aur-A in ccRCC tissues (T) and normal adjacent tissues (N), and ccRCC cell lines. **C** Expression value of Aur-A gene was higher and related to a worse overall survival in ccRCC. Bar graph showing expression values of Aur-A gene using the study dataset, and data from http://ualcan.path.uab.edu. Over survival was indicated by Kaplan–Meier survival analysis (log-rank test, *p* < 0.001). **D** SKP2 was positively correlated with Aur-A in ccRCC human tissues. ccRCC tissue microarrays were stained with Aur-A and SKP2, and then photographed (Scale bars, 200 and 50 μm). Association analysis of Aur-A and SKP2 in ccRCC by using SPSS software. The protein levels were normalized to GAPDH. ns no significance; **p < 0.01, ***p < 0.001; *n* = 3 independent experiments; two-tailed paired Student’s *t* test or one-way ANOVA with Dunnett’s test.

**Fig. 3:**
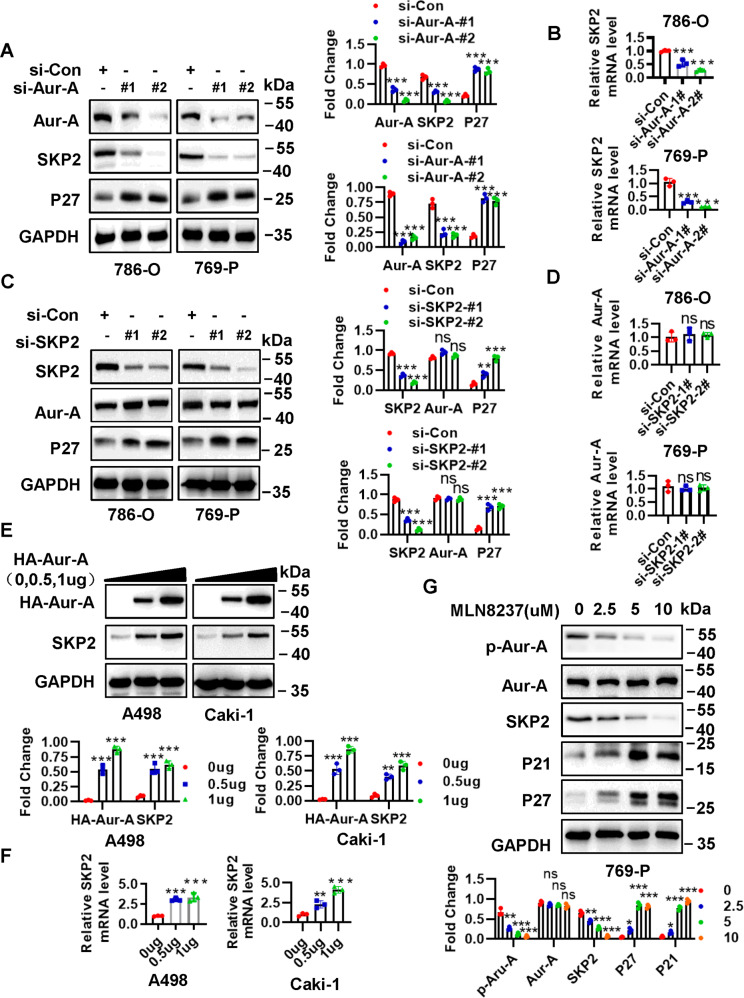
Aur-A regulates SKP2 by its kinase activity. **A**, **B** Silencing of Aur-A decreased protein expression and mRNA of SKP2 in ccRCC cell lines. 786-O and 769-P ccRCC cell lines were transfected with siRNAs targeting Aur-A, followed by IB (**A**) and qPCR **B** analysis, respectively. **C**, **D** Depletion of SKP2 had no effect on neither protein expression nor mRNA of Aur-A in ccRCC cell lines. 786-O and 769-P ccRCC cell lines were transfected with siRNAs targeting SKP2, followed by IB (**C**) and qPCR **D** analysis, respectively. **E**, **F** Overexpression Aur-A increased protein expression and mRNA of SKP2 in a dose-dependent manner. A498 and Caki-1 ccRCC cell lines were transfected with vector or HA-Aur-A (0, 0.5, 1 μg), followed by IB (**E**) and qPCR (**F**) analysis, respectively. **G** Aur-A inhibitor MLN8237 decreased protein expression of both p-Aur-A and SKP2 in ccRCC cells. 769-P cells were incubated with indicated doses of MLN8237 (0, 2.5, 5, and 10 μM) for 48 h, followed by IB analysis. The protein levels were normalized to GAPDH. ns no significance; *p < 0.05, **p < 0.01, ***p < 0.001; *n* = 3 independent experiments; one-way ANOVA with Dunnett’s test.

As it has been previously identified SPOP, as an adapter protein for E3 ubiquitin ligase, is a substrate of Aur-A, and Aur-A could phosphorylate SPOP, causing its ubiquitylation [28], thus we further wondered whether SKP2 E3 ligase as same as SPOP is regulated by Aur-A. First, correlation analysis revealed that Aur-A was largely correlated in a positive manner with SKP2 (Fig. 2A). In ccRCC tumor tissues and cell lines, protein level of Aur-A was highly expressed (Fig. 2B), and as well as high expression of Aur-A was related to a worse overall patient survival by TCGA database and Kaplan–Meier survival analysis (Fig. 2C). Furthermore, in 85 ccRCC TMA samples, the expression of Aur-A was positively correlated with the expression of SKP2, which was demonstrated by the high Aur-A staining with high SKP2 staining in the same samples (*p* = 0.001, *r* = −0.572), while the low Aur-A staining group was associated with low staining group of SKP2 (Fig. 2D). Having detected a physical interaction between Aur-A and SKP2, we performed an siRNA-based knockdown experiment in 786-O and 769-P ccRCC cells. We found that endogenous protein level of SKP2 and its mRNA were significantly down-regulated upon Aur-A knockdown (Fig. 3A, B). But SKP2 depletion using siRNA in 786-O and 769-P cell lines had no effect on protein expression and mRNA level of Aur-A (Fig. 3C, D). Both SKP2 depletion and Aua-A depletion could increase p27 protein (Fig. 3A, C). Thus, we speculated that regulation of SKP2 expression might be mediated by Aur-A. When HA-Aur-A was transfected, a dose-dependent accumulation of endogenous SKP2 protein was detected in A498 and Caki-1 ccRCC cells (Fig. 3E). Likewise, overexpression of Aur-A could also lead to the accumulation of SKP2 mRNA in a dose manner (Fig. 3F). These results confirmed that Aur-A-mediated SKP2 regulation was mainly achieved in a transcriptional manner. We then sought to determine whether Aur-A mediates SKP2 via its kinase activation. We treated 769-P cells with MLN8237, a specific inhibitor of Aurora-A kinase, and confirmed that MLN8237 inhibited Aur-A by reducing its autophosphorylation, leading to the reduction of SKP2 in a dose-dependent manner (Fig. 3G). In addition, our data showed that inactivation of Aur-A caused the increase of p21 and p27. Collectively, Aur-A regulated SKP2 protein level upon its kinase activation in ccRCC.

### Aur-A regulates SKP2 by mediating FOXO3A

**Fig. 4:**
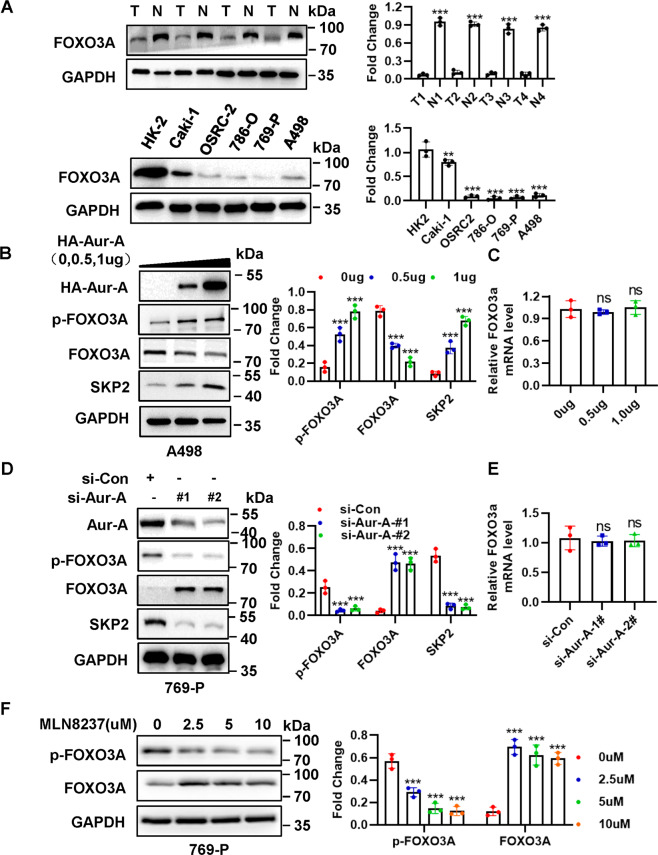
Aur-A regulates SKP2 by mediating FOXO3A. **A** Basal level of Aur-A in ccRCC tissues (T) and normal adjacent tissues (N), and ccRCC cell lines. **B**, **C** Overexpression of Aur-A induced reduction of FOXO3A and increase of p-FOXO3A, but did not affect mRNA of FOXO3A. A498 cells were transfected with vector or HA-Aur-A (0, 0.5, 1 μg), and followed by IB (**B**) and qPCR **C** analysis, respectively. **D**, **E** silencing of Aur-A increased FOXO3A and decreased p-FOXO3A, but did not affect mRNA of FOXO3A. 769-P cells were transfected siRNAs targeting Aur-A (siRNA-Aur-A-1# and siRNA-Aur-A-2#), and followed by IB (**D**) and qPCR **E** analysis, respectively. **F** Aur-A inhibitor MLN8237 decreased FOXO3A protein and increased p-FOXO3A protein in ccRCC cells. 769-P cells were incubated with indicated doses of MLN8237 (0, 2.5, 5, and 10 μM) for 48 h, followed by IB analysis. The protein levels were normalized to GAPDH. ns no significance; **p < 0.01, ***p < 0.001; *n* = 3 independent experiments; two-tailed paired Student’s *t* test or one-way ANOVA with Dunnett’s test.

Given that the protein and mRNA levels of SKP2 were elevated or reduced by Aur-A overexpression or silencing, respectively (Fig. 3). And Wu et al. reported that FOXO3A transcription factor was a negative regulator of SKP2 [29], therefore we speculated Aur-A regulated SKP2 by mediating FOXO3A. First, we found that FOXO3A was less expressed in ccRCC tumor tissues and cell lines (Fig. 4A), and negatively correlated with Aur-A and SKP2 expression (Figs. 1C and 2C). Then, we further transfected HA-Aur-A into A498 ccRCC cells and found the reduction of FOXO3A and increase of p-FOXO3A (Fig. 4B). Conversely, we performed an siRNA-based knockdown experiment in 769-P ccRCC cells and found the increase of FOXO3A and reduction of p-FOXO3A upon Aur-A knockdown (Fig. 4D). However, overexpression or knockdown of Aur-A had no effect on the mRNA level of FOXO3A (Fig. 4C, E). Importantly, Aur-A inhibitor MLN8237 led to the increase of FOXO3A and reduction of p-FOXO3A in a dose-dependent manner (Fig. 4F). Collectively, our data showed that Aur-A regulated SKP2 by mediating FOXO3A.

### FOXO3A ubiquitylation and degradation by SKP2 mediating depends on the kinase activity of Aur-A

**Fig. 5:**
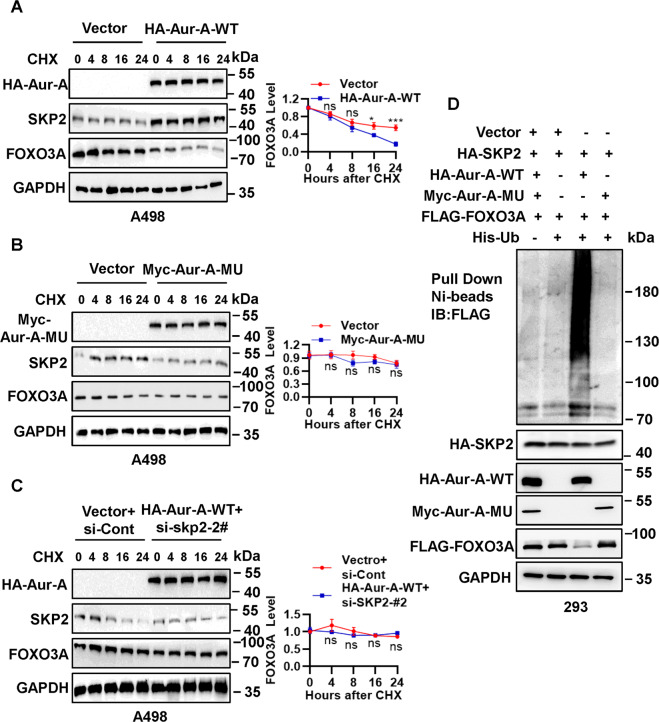
FOXO3A ubiquitylation and degradation by SKP2 mediating depends on the kinase activity of Aur-A. **A**, **B** Overexpression of wild-type Aur-A significantly shortened the protein half-life of endogenous FOXO3A and enhanced SKP2 protein in ccRCC cells, but its mutant failed. After A498 cells were transfected with HA- Aur-A-WT (**A**) or myc-Aur-A-MU (**B**) for 48 h, cells were switched to fresh medium (10% FBS) containing cycloheximide (CHX) and incubated for indicated time periods before being harvested for IB. **C** Simultaneously transfected with Aur-A-WT and siRNA-SKP2, which had no effects on FOXO3A half-life. A498 cells were transfected with vector or HA-Aur-A-WT, along with transfection of siRNAs targeting SKP2 (siRNA-SKP2-2#), cells were switched to fresh medium (10% FBS) containing CHX and incubated for indicated time periods before being harvested for IB. **D** SKP2 promoted ubiquitylation of FOXO3A by dependent on kinase activity of Aur-A. 293 cells were transfected with indicated plasmids, lysed under denatured condition at 6 M guanidinium solution, followed by Ni-beads pull-down. Washed beads were boiled for IB to detect ubiquitylation of exogenous FOXO3A. The protein levels were normalized to GAPDH. ns no significance; *p < 0.05, ***p < 0.001; *n* = 3 independent experiments; two-tailed unpaired Student’s *t* test.

It is well-established that phosphorylation of a substrate is prerequisite in most cases for its binding to E3 ubiquitin ligase protein for subsequent ubiquitylation and degradation [30]. We further confirmed that the levels of FOXO3A and SKP2 are negatively or positively regulated by kinase activity of Aur-A, as evidenced by FOXO3A half-life reduction and SKP2 accumulation upon wild-type Aur-A (Aur-A-WT) overexpression (Fig. 5A), and by FOXO3A half-life prolong and SKP2 reduction upon Aur-A kinase-dead (D281) mutant (Aur-A-MU), respectively (Fig. 5B). When simultaneously transfected with Aur-A-WT and siRNA-SKP2 (siRNA-SKP2-#2), it had no effect on FOXO3A half-life (Fig. 5C). Some studies have shown that the ubiquitination and degradation of FOXO3A are also regulated by SKP2 [31, 32]. Based on this, we next used classical in vivo ubiquitination analysis to determine whether SKP2 will promote FOXO3A ubiquitination and degradation by relying on Aur-A kinase activity. The in vivo ubiquitylation assay results showed that SKP2 significantly promoted ubiquitylation of exogenously expressed FOXO3A by dependent on wild-type Aur-A, but not its Aur-A mutant (Fig. 5D). Taken together, our results supported the notion that SKP2 promoted FOXO3A ubiquitylation for subsequent degradation by dependent on kinase activity of Aur-A.

### Combination of MLN8237 and SZL P1-41 shows synergistic lethality in vitro

**Fig. 6:**
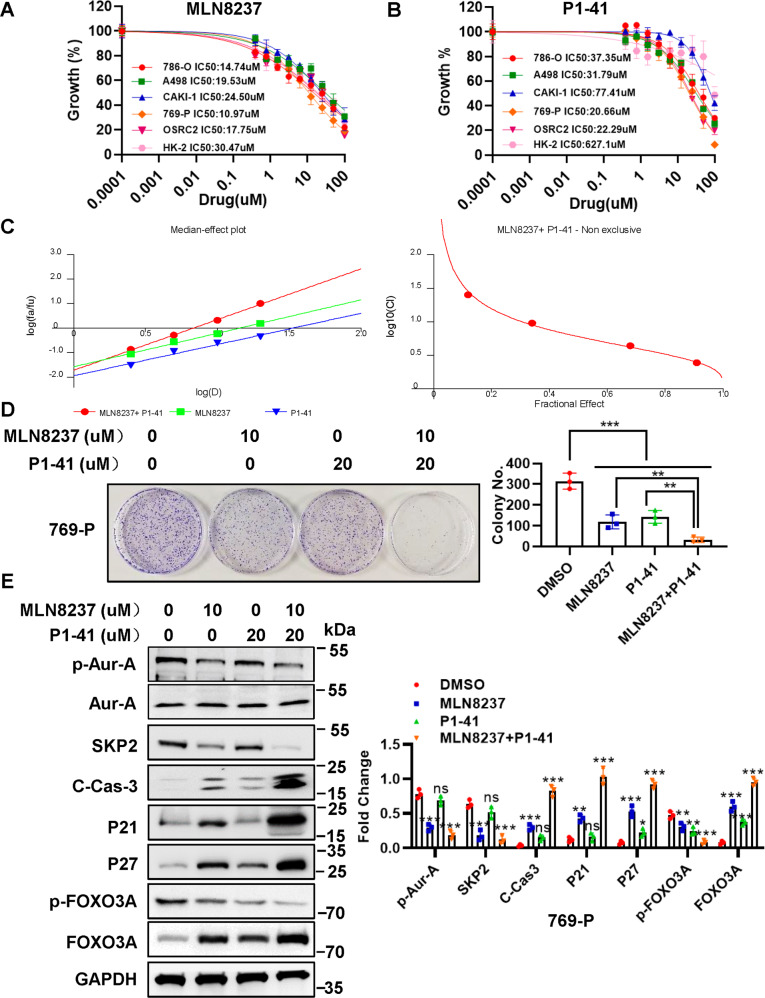
Combination of MLN8237 and SZL P1-41 shows synergistic lethality in vitro. **A**, **B** IC50 values of MLN8237 and SZL P1-41 in five ccRCC cell lines and HK-2 cell line. Each cell line was treated with the indicated concentrations of MLN8237 (0, 0.39, 0.78, 1.56, 3.125, 6.25, 12.5, 25, 50, and 100 μM) (**A**) or SZL P1-41 (0, 0.39, 0.78, 1.56, 3.125, 6.25, 12.5, 25, 50, and 100 μM) (**B**) for 48 h, followed by the CCK-8 assay. The IC50 of the two compounds were determined by GraphPad Prism5 software. **C** MLN8237 and SZL P1-41 showed synergistic effects in 769-P ccRCC cell line. CI-effect plots and median effect plots were generated using CalcuSyn software. The points a, b, c, and d represent CI values for the combinations 1.25, 2.5, 5, and 10 μM MLN8237 with 2.5, 5, 10, and 20 μM SZL P1-41 in a constant ratio, respectively. **D** Combination of MLN8237 and SZL P1-41 inhibited clonogenic ability in ccRCC cells. 769-P cells were treated with MLN8237 (10 μM) or SZL P1-41 (20 μM) alone or combination of the two compounds, followed by the colony formation assay. **E** Dual inhibition of Aur-A and SKP2 significantly increased protein levels of C-Cas-3, p21, p27, FOXO3A and decreased p-Aur-A, SKP2 and p-FOXO3A in ccRCC cells. 769-P cells were treated with MLN8237 (10 μM) or SZL P1-41 (20 μM), or combination of the two compounds, followed by the IB assay with indicated antibodies. The protein levels were normalized to GAPDH. ns no significance; **p < 0.01, ***p < 0.001; *n* = 3 independent experiments; one-way ANOVA with Dunnett’s test.

We found that Aur-A kinase inhibitor MLN8237 could reduce SKP2 expression by enhancing FOXO3A (Fig. 4). Therefore, we supposed that a combination of Aur-A inhibitor MLN8237 and SKP2 inhibitor SZL P1-41 might have dual inhibition effects on ccRCC cells. SZL P1-41 is a specific Skp2 inhibitor, binds to the F-box domain of Skp2 to prevent Skp1 association and Skp2 SCF complex formation. To determine the effects of dual inhibition of SKP2 and Aur-A, we first identified the IC50 of Aur-A kinase inhibitor MLN8237 and SKP2 inhibitor SZL P1-41 in different ccRCC cell lines. Both MLN8237 (IC50 = 10.97 μM) and SZL P1-41 (IC50 = 20.66 μM) monotherapy inhibited cell proliferation significantly in 769-P and showed less effects in Caki-1 cells (Fig. 6A, B). We then chose the 769-P ccRCC cell line for the following combination experiments. Both CalcuSyn software and Jin’s formula were used as previously described to determine the synergy of the two agents. 769-P cells were cultured with the combination of two drugs at different doses but in a constant ratio (MLN8237 to SZL P1-41: 1.25–2.5, 2.5–5, 5–10 and 10–20 μM, respectively) for 48 h. The combination of 1.25 μM MLN8237 with 2.5 μM SZL P1-41 in 769-P cells inhibited cell proliferation by 12.0%, compared with monotherapy of MLN8237 by 8.0% or SZL P1-41 by 3.0%, indicating synergism (CI = 1.527; *Q* = 1.12; Fig. 6C). Escalating doses, i.e., co-treatment with 2.5 μM MLN8237 with 5 μM SZL P1-41 (CI = 1.113; *Q* = 1.14) or 5 μM MLN8237 with 10 μM SZL P1-41 (CI = 0.891; *Q* = 1.20) or 10 μM MLN8237 with 20 μM SZL P1-41 (CI = 0.397; *Q* = 1.21), showed synergetic effects in 769-P cells (Fig. 6C). Furthermore, a combination of MLN8237 (10 μM) with SZL P1-41 (20 μM) significantly inhibited the clonogenic survival in 769-P ccRCC cells (MLN8237 or SZL P1-41 vs. MLN8237 + SZL P1-41: *p < 0.05, **p < 0.01; Fig. 6D), indicating that the combination of the two agents significantly inhibited cell growth, which was further demonstrated by the increase of p21, p27 and apoptosis-related protein C-Cas-3 (Fig. 6E). In addition, MLN8237 and SZL P1-41 combination also significantly increased FOXO3A and decreased p-FOXO3A, p-Aur-A, and SKP2 (Fig. 6E).

### Combination of MLN8237 and SZL P1-41 shows synergistic lethality in vivo

**Fig. 7:**
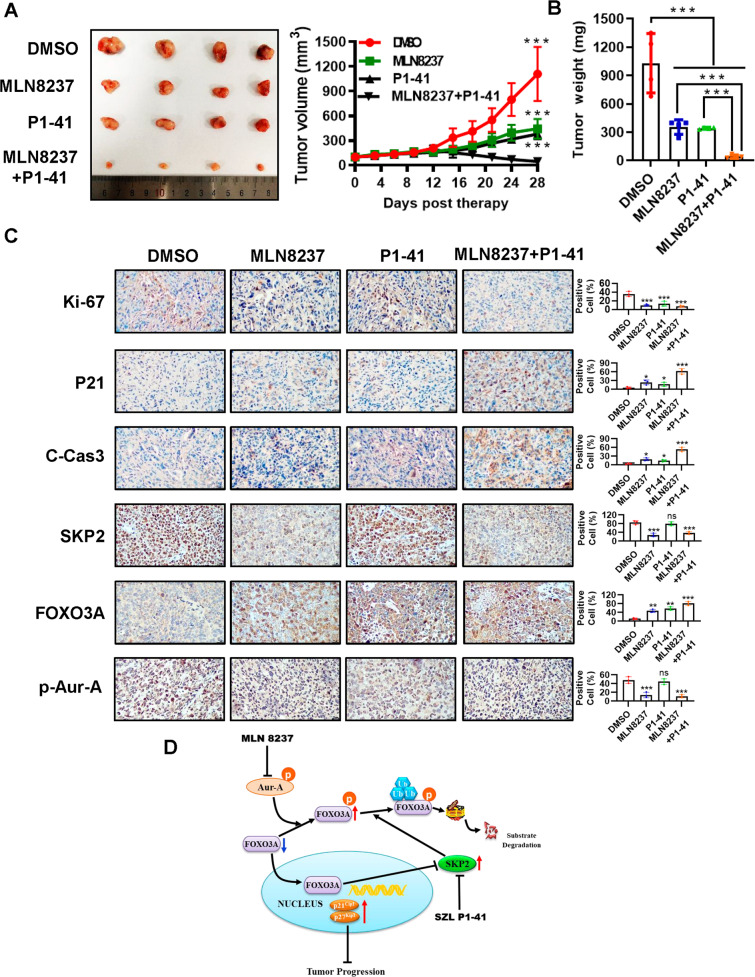
MLN8237 and SZL P1-41 synergistically inhibit ccRCC tumor growth in vivo. **A**, **B** Synergistic antitumor activity of MLN8237 and SZL P1-41 in the 769-P xenograft model. 769-P ccRCC cells were injected subcutaneously into right flank side of nude mice. The nude mice were randomized when the tumor size reached 100 mm^3^ and were treated as follows: DMSO, *n* = 4; MLN8237 (40 mg/kg/day of every day for 4 weeks), *n* = 4; SZL P1-41 (80 mg/kg/day of every day for 4 weeks), *n* = 4; MLN8237 + rapamycin, *n* = 4. The tumors were harvested and tumor growth was monitored (**A**), and tumor weight was measured (**B**). **C** Immunohistochemical staining of xenograft tumor tissues. Tumor tissues from four groups of nude mice were fixed, sectioned, and stained with indicated antibodies. Scale bars: 20 μm. **D** Schematic model depicting the mechanisms of action underlying Aur-A/FOXO3A/SKP2 axis promoted ccRCC cell progression and the combination of Aur-A inhibitor MLN8237 and SKP2 inhibitor SZL P1-41 showed a synergistic lethality. ns no significance; *p < 0.05, **p < 0.01, ***p < 0.001; *n* = 3 independent experiments; two-tailed unpaired Student’s *t* test or one-way ANOVA with Dunnett’s test.

We next validated the above in vitro findings by using an in vivo xenograft model. 769-P ccRCC xenograft model was established, and then treated with DMSO, MLN8237 (40 mg/kg/day, every day, p.o.), SZL P1-41 (80 mg/kg/day, every day, i.p.) and MLN8237 + SZL P1-41. Consistent with the in vitro results, the average tumor size and tumor weight at the end of experiment (treatment with 28 days) were significantly lower in the combination MLN8237 and SZL P1-41 group (MLN8237 or SZL P1-41 vs. MLN8237 + SZL P1-41, ***p < 0.001; Fig. 7A, B). Likewise, IHC staining of tumor tissues revealed that, compared with MLN8237 or SZL P1-41 single-agent treatment, a combination of the two agents more significantly inhibited tumor growth (decrease of Ki-67 and increase of p21) and induced apoptosis (increase of C-Cas-3) (Fig. 7C). Moreover, the combination of MLN8237 and SZL P1-41 caused a crease of FOXO3A and a decrease of SKP2 and p-Aur-A (Fig. 7C). Collectively, the results from both in vitro cell culture and in vivo xenograft models coherently demonstrated that the combination of MLN8237 and SZL P1-41 more significantly repressed ccRCC cell growth than single-agent treatment, with less effect on normal tissues.

## Discussion

Emerging evidences have reported that SKP2 is frequently overexpressed in prostate cancer [10], melanoma [11], nasopharyngeal carcinoma [12], and breast cancer [13], and associated with poor prognosis by enhancing tumor progression [7]. However, biological function of SKP2 in ccRCC is rarely known. Here we showed that SKP2 also acted as an oncogene in ccRCC with following lines of supporting evidence. (1) SKP2 protein is highly expressed in ccRCC tumor tissues and cells; (2) overexpression of SKP2 enhances cell proliferation and clone-forming ability of ccRCC, while SKP2 depletion exhibits the opposite effect; (3) high levels of SKP2 predicts a worse patient survival, whose results are consistent with previous studies [15, 16]. Collectively, functional studies have revealed that SKP2 promotes cell growth of ccRCC and is associated with poor prognosis.

Most studies have reported that Aur-A is involved in the growth regulation of various cancers and associated with drug resistance, and SPOP E3 ubiquitin ligase was identified as a substrate of Aur-A [28]. As a E3 ubiquitin ligase is that, SKP2 has never been reported to be associated with Aur-A before. In this study, we identified Aur-A regulated SKP2 by mediating FOXO3A. Our conclusion is supported by the following lines of evidence: (1) SKP2 is positively correlated with Aur-A in ccRCC tissues and cells, and protein and mRNA levels of SKP2 are elevated or reduced by Aur-A overexpression or silencing, respectively; (2) FOXO3A is a negative transcription factor for SKP2 [31, 32], and FOXO3A ubiquitylation and degradation by SKP2 mediating depends on the kinase activity of Aur-A; (3) the combination of Aur-A inhibitor MLN8237 and SKP2 inhibitor SZL P1-41 show a synergistic tumor growth inhibition in vivo and in vitro of ccRCC models. Collectively, functional studies have revealed that growth-promoting effect of SKP2 is mediated to promote FOXO3A ubiquitylation and degradation by dependent on kinase activity of Aur-A.

Given its potential role in disease progression, Aur-A is an attractive target for cancer therapy. At present, numerous Aur-A inhibitors have been developed, such as Alisertib (MLN8237). MLN8237 has been shown suppression of activity against a broad range of tumor types. Therefore, single-agent Alisertib has been used for multiple cancers in clinical trials. But it still exhibits poor efficacy and is limited by the modest side effects. For example, Alisertib shows a modest anti-leukemic activity [33]. Overactivation of mTOR also commonly occurs in multiple cancer types [34, 35], some studies revealed that Aur-A/ERK1/2/mTOR formed an oncogenic cascade in triple-negative breast cancer (TNBC), and double inhibition of Aur-A and mTOR showed significant synergistic effects in TNBC models. Here, we also found that dual inhibition of Aur-A and SKP2, compared with monotherapy, inhibited more cell proliferation and survival in vitro and in vivo in ccRCC models. Therefore, Aur-A deregulation and subsequent activation of the SKP2 pathway plays an important role in ccRCC tumorigenesis and provide a supporting justification for clinical trials to evaluate combined Aur-A and SKP2 inhibitors to enhance the anti-ccRCC effects observed with single-agent inhibitor alone.

In conclusion, we reveal that overexpression of SKP2 enhances cell proliferation of ccRCC, while SKP2 depletion exhibits the opposite effect. SKP2 is positively correlated with Aur-A in ccRCC tissues and cells, and its protein and mRNA levels are elevated or reduced by Aur-A overexpression or silencing, respectively. It is further found that Aur-A also causes an increase phosphorylation of FOXO3A, which its ubiquitylation and degradation by SKP2 mediating depends on the kinase activity of Aur-A. Collectively, Aur-A/FOXO3A/SKP2 axis can promote ccRCC cell progression and the combination of Aur-A inhibitor MLN8237 and SKP2 inhibitor SZL P1-41 shows a synergistic lethality in vivo and in vitro, indicating a potential new therapeutic strategy for ccRCC (Fig. 7D).

## Materials and methods reagents

MG-132 (#HY-13259), Cycloheximide (#HY-12320), MLN8237 (#HY-10971), and SZL P1-41(#HY-100237) were purchased from MedChemExpress, and dissolved in dimethyl sulfoxide (DMSO) (#N182, Amresco) and stored at −20 °C.

### ccRCC tissue samples and cell lines

To detect the protein level of VHL, SKP2, Aua-A and FOXO3A in ccRCC tissue, total of four ccRCC tissue samples and non-tumor adjacent tissues samples were derived from surgical specimens and pathologically confirmed as ccRCC from the Second Affiliated Hospital of Third Military Medical University from June 2020 to May 2021. All procedures were conducted with the approval of the Ethics Committee of Second Affiliated Hospital of Third Military Medical University. All patients enrolled in the study signed informed consent according to their preference.

The ccRCC cell lines (A498, Caki-1, OSRC-2, 786-O, and 769-P) and the immortalized epithelial renal cell line (HK-2) were obtained from the American Type Culture Collection (ATCC). 786-O 769-P, and OSRC-2, cells were maintained in RPMI-1640 medium (Gibco, #21870076). A498 cells grown in DMEM (Gibco, #11965092). The Caki-1 cells were cultured in McCoy’s 5A medium (Gibco, #16600082). Each media was supplemented with 10% fetal bovine serum and 1% penicillin-streptomycin. The HK-2 cell line was cultured in KSFM medium (Gibco, #10744019). And all cells cultured at 37 °C in a humidified atmosphere containing 5% CO_2_. All cell lines were tested and free of mycoplasma contamination.

### RNA isolation, reverse-transcription, and qPCR

Total RNA from cells were isolated using Trizol-Chloroform method and then transcribed into cDNA using reverse-transcription kit (Takara, Japan) with an oligo (dT) 20 bp primer. RT-qPCR was performed by using the SYBR green reagent (Takara, Japan) on Real-Time PCR System (Thermo Fisher, USA). The 2^−ΔΔCt^ method was used to quantify the data, and GAPDH was used as the housekeeping gene. The following forward (F) primers and reverse (R) primers were used: SKP2 (forward 5′-GATGTGACTGGTCGGTTGCTGT-3′ and reverse 5′-GAGTTCGATAGGTCCA TGTGCTG-3′), Aur-A (forward 5′-TGGAATATGCACCACTTGGA-3′ and reverse 5′-GGCATTTGCCAATTCTGTTA-3′), FOXO3A (forward 5′-GCTCAGGAGCTG ATGATCCA-3′; reverse 5′-GCGCTTGATCCTCGTGTAG-3′) and GAPDH (forward 5′-TGACTTCAACAGCGACACCCA-3′ and reverse 5′-CACCCTGTTGCTGTAGC CAAA-3′). Each experiment was conducted in triplicate.

### Immunoblotting and immunohistochemistry

Immunoblotting (IB) analysis was performed as previously described [36]. The following primary antibodies were used in this study: anti-Aur-A (cell signaling, #91590; 1:1000), anti-Phospho-Aur-A (Thr288) (cell signaling, #3079; 1:1000), anti-SKP2 (cell signaling, #2652; 1:1000), anti-VHL (cell signaling, #68547; 1:1000), anti-FOXO3A (cell signaling, #12829; 1:1000), anti-Phospho-FOXO3A (cell signaling, #4691; 1:1000), p21 (Proteintech, 10355-1-AP; 1:1000), p27 (Proteintech, 25614-1-AP; 1:1000), Cleavage Caspase-3 (Asp175) (5A1E) (cell signaling, #9661; 1:1000), FLAG (Sigma-Aldrich, #F4042; 1:1000), HA-tag (Proteintech, 51064-2-AP; 1:1000), Myc-tag (Proteintech, 16286-1-AP; 1:1000) and GAPDH (cell signaling, #5174; 1:1000). GAPDH was used as an internal control.

Immunohistochemical (IHC) staining of human ccRCC tissues and mice tumors were performed as described previously [37]. Briefly, after deparaffinization, rehydration, antigen retrieval and blocking, the tissue slides were incubated overnight at 4 °C with indicated antibodies. The following primary antibodies were used: anti-SKP2 (Proteintech, 15010-1-AP; 1:200), anti-Aur-A (Proteintech, 10297-1-AP; 1:200), anti-Ki-67 (Cell Signaling, #9449, 1:1000), anti-p21 (Proteintech, 10355-1-AP, 1:500) and anti-Cleaved-capase-3 (Asp175) (5A1E) (Cell Signaling, #9661,1:500), anti-FOXO3aA (cell signaling, #469112829; 1:1000) and anti- Phospho-Aurora-A (Thr288) (Cell Signaling, #3079, 1:200). IHC results were scored by taking into account the percentage of tumor tissue with indicated protein staining (0–100%) by ImageJ software and the intensity of the staining (0, negative; 1, weak; 2, moderate; 3, strong) conducted by two independent pathologists.

### siRNAs, plasmids, and transfection

Plasmids of HA-Aur-A-WT, Myc-Aur-A-MU(D281A), and FLAG-SKP2 were obtained from Dr. Jie Xu from Third Military Medical University. The targeting plasmids were delivered into A498 or Caki-1 cells using the Lipofectamine 2000 transfection reagent (Invitrogen, USA, #11668019) according to the manufacturer’s instructions. siRNA designed to target SKP2 or Aur-A were purchased from Santa Cruz, including siRNA-SKP2-#1 (5′-GUGAUAGUGUCAUGCUAAATT-3′ and 5′-UUUAGCAUGACACUAUCACTT-3′), siRNA-SKP2-#2 (5′-GUACAGCACAUGGACCUAUTT-3′ and 5′-AUAGGUCCAUGUGCUGUACTT-3′), si-Aur-A-#1 (5′-GGAUACUGCUUGUUACUUAUU-3′ and 5′-UAAGUAACAAGCAGUAUCCUA-3′) and si-Aur-A-#2 (5′-GCUCAGAAGAGAAGUAGAAAU-3′ and 5′-UUCUACUUCUCUUCUGAGCUG-3′), Scrambled control siRNA sequences were 5′-CGUAUGCGCGUACUCUAAUTT-3′ and 5′-TTGCAUACGCGCAUGAGAUUA-3′. For transient transfection, control, SKP2 siRNA (#1 and #2), or Aur-A siRNA (#1 and #2) were mixed with Lipofectamine 2000 and then added to cell culture medium in 786-O or 769-P cells according to the manufacturer’s instructions.

### Colony formation assay

Briefly, the transfected 786-O and A498 cells were seeded into 60-mm dishes (Corning, NY, USA) at a density of 2 × 10^3^ cells/well, respectively, followed by incubation at 37 °C for 10–14 days. 769-P cells were seeded into 60-mm dishes at a density of 2 × 10^3^ cells/well. After 24 h, cells were treated with MLN8237 (10 μM), SZL P1-41 (20 μM), or MLN8237 + SZL P1-41 for 48 h, which was then removed and replaced by complete medium, followed by incubation at 37 °C for 10–14 days. The colonies were fixed with 4% paraformaldehyde for 15 min, stained with crystal violet at room temperature for 30 min, and then counted [38]. Each experiment was conducted in triplicate.

### Cell viability assays

The Cell Counting Kit-8 assay (#HY-K0301, MCE) was used to assess the cell viability. The concentration of the transfected 786-O and A498 cells were adjusted to 2 × 10^3^ cells/well, and then were seeded into 96-well plates in triplicate. At different time after cell plating, the cell viability was determined by measuring the optical density at 450 nm. The concentration of ccRCC cells and HK-2 cells were adjusted to 2 × 10^3^ cells/well, and the cells were seeded into 96-well plates, followed by 24 h of culture at 37 °C in an atmosphere with 5% CO_2_, then were treated with various concentrations of MLN8237 or SZL P1-41, and maintained in culture for 48 h. After removing the culture medium, the cell viability was determined. The half-maximal inhibitory concentration (IC50) value is a critical index of the dose-response curve. Prism statistical software (GraphPad, San Diego, CA, USA) was used to calculate the IC50 values and to plot dose-response curves. Each experiment was conducted in triplicate.

### Half-life analysis

After gene manipulation, 20 μg/ml cycloheximide (CHX, MCE) was added to the cell medium to inhibit new protein synthesis. At the indicated time points, cells were harvested, lysed and subjected to IB analysis. Each experiment was conducted in triplicates.

### The in vivo ubiquitylation

To detect ubiquitination of exogenous FOXO3A, 293T cells were co-transfected with FLAG-FOXO3A, HA-SKP2, His-Ub, and HA-Aur-A-WT or Myc-Aur-A-MU. Mock vector was used as a control for ubiquitination assay. In vivo ubiquitylation assays were performed as previously described using Ni-beads pull-down [30]. And cells were treated with MG132 (10 μM) for another 4 h before lysed. The beads were boiled and the pull-down proteins were resolved with anti-HA or anti-FLAG antibody by subsequently IB assay. Each experiment was conducted in triplicate.

### Tissue microarray

Tissue microarray was purchased from avilabio (Xi’an, China), which provided chip information and completed IHC staining of the chips. IHC staining was performed on 85 cases of ccRCC tumor tissues to detect Aur-A and SKP2. The stained slides were observed under a microscope, and images were acquired and quantitatively classified based on staining intensity, as described previously [39].

### In vivo xenograft model

All animal experiments were carried out according to a protocol approved by the Laboratory Animal Welfare and Institutional Animal Care and Use Committee of Model Animal Research Center of Army Medical University (Third Military Medical University) (Chongqing, China). A total of sixteen BALB/C nude mice (nu/nu; female; aged, 4–6 weeks; body weight, 18–20 g) were purchased from Beijing Vital River Laboratory Animal Technology Co., Ltd. and randomly divided into four groups. In total, 2 × 10^6^ 769-P cells were mixed 1:1 with matrigel (BD Biocoat #354230) in a total volume of 0.2 ml, and then injected subcutaneously into right flank side of mice. When the tumor volume (TV) reached 100^3^, which were randomized and treated with vehicle, MLN8237 (40 mg/kg/day, every day, p.o.), SZL P1-41 (80 mg/kg/day, every day, i.p.) and MLN8237 + SZL P1-41. Nude mice were followed up for tumor size, and body weight. After 4 weeks of treatment, mice were euthanized by carbon dioxide asphyxiation (30% vol/min). Tumors were resected, weighed, and frozen or fixed in formalin and paraffin-embedded for IHC studies. The growth of tumor was measured twice a week and average TV was calculated according to the equation: TV = (*L* × *W*^2^)/2, where *L* is the length and *W* is the width of the tumor.

### Statistical analysis

All data were expressed as the mean ± standard deviation and statistical charts were prepared using GraphPad Prism 7.0 software (GraphPad Software, Inc.) and SPSS 20.0 (IBM Corporation). Welch’s test was conducted for differential expression of functional mRNA in UALCAN (http://ualcan.path.uab.edu/). Log-rank test was implemented in GEPIA (http://gepia.cancer-pku.cn/) for comparison of overall survival curves, which displayed as Kaplan–Meier plot. Transcripts per million values and Student’s *t* tests were employed to calculate the significance of gene expression divergence between categories in GEPIA (http://gepia.cancer-pku.cn/). Both CalcuSyn software and Jin’s formula were used to evaluate the synergistic effects of drug combinations as described previously [40, 41]. The Chi-square test or Fisher’s exact test was performed to analyze the correlation between the expression level of SKP2 and Aur-A. Differences between two groups were analyzed using an unpaired Student’s *t* test, while the expression of some genes (VHL, SKP2, Aur-A, FOXO3A) in tumor tissues and matched normal tissues was compared using a paired Student’s *t* test. Comparisons of multiple groups were performed using one-way ANOVA followed by Dunnett’s test. *p* < 0.05 is considered significant in this entry (*p < 0.05; **p < 0.01; ***p < 0.001).

## Supplementary information

aj-checklist

Original Data File

## Data Availability

All datasets generated and analyzed during this study are included in this published article and its Supplementary Information files. Additional data are available from the corresponding author on reasonable request.

## References

[1] Barata PC, Rini BI (2017). Treatment of renal cell carcinoma: current status and future directions. CA Cancer J Clin.

[2] Hsieh JJ, Purdue MP, Signoretti S, Swanton C, Albiges L, Schmidinger M (2017). Renal cell carcinoma. Nat Rev Dis Prim.

[3] Flanigan RC, Salmon SE, Blumenstein BA, Bearman SI, Roy V, McGrath PC (2001). Nephrectomy followed by interferon alfa-2b compared with interferon alfa-2b alone for metastatic renal-cell cancer. N Engl J Med.

[4] McDermott DF, Regan MM, Clark JI, Flaherty LE, Weiss GR, Logan TF (2005). Randomized phase III trial of high-dose interleukin-2 versus subcutaneous interleukin-2 and interferon in patients with metastatic renal cell carcinoma. J Clin Oncol.

[5] Sun M, Lughezzani G, Perrotte P, Karakiewicz PI (2010). Treatment of metastatic renal cell carcinoma. Nat Rev Urol.

[6] Bai C, Sen P, Hofmann K, Ma L, Goebl M, Harper JW (1996). SKP1 connects cell cycle regulators to the ubiquitin proteolysis machinery through a novel motif, the F-box. Cell.

[7] Craig KL, Tyers M (1999). The F-box: a new motif for ubiquitin dependent proteolysis in cell cycle regulation and signal transduction. Prog Biophys Mol Biol.

[8] Nakayama KI, Nakayama K (2005). Regulation of the cell cycle by SCF-type ubiquitin ligases. Semin Cell Dev Biol.

[9] Wang H, Cui J, Bauzon F, Zhu L (2010). A comparison between Skp2 and FOXO1 for their cytoplasmic localization by Akt1. Cell Cycle.

[10] Wang Z, Gao D, Fukushima H, Inuzuka H, Liu P, Wan L (2012). Skp2: a novel potential therapeutic target for prostate cancer. Biochim Biophys Acta.

[11] Rose AE, Wang G, Hanniford D, Monni S, Tu T, Shapiro RL (2011). Clinical relevance of SKP2 alterations in metastatic melanoma. Pigment Cell Melanoma Res.

[12] Fang FM, Chien CY, Li CF, Shiu WY, Chen CH, Huang HY (2009). Effect of S-phase kinase-associated protein 2 expression on distant metastasis and survival in nasopharyngeal carcinoma patients. Int J Radiat Oncol Biol Phys.

[13] Radke S, Pirkmaier A, Germain D (2005). Differential expression of the F-box proteins Skp2 and Skp2B in breast cancer. Oncogene.

[14] Davidovich S, Ben-Izhak O, Shapira M, Futerman B, Hershko DD (2008). Over-expression of Skp2 is associated with resistance to preoperative doxorubicin-based chemotherapy in primary breast cancer. Breast Cancer Res.

[15] Chan CH, Li CF, Yang WL, Gao Y, Lee SW, Feng Z (2012). The Skp2-SCF E3 ligase regulates Akt ubiquitination, glycolysis, herceptin sensitivity, and tumorigenesis. Cell.

[16] Han F, Li CF, Cai Z, Zhang X, Jin G, Zhang WN (2018). The critical role of AMPK in driving Akt activation under stress, tumorigenesis and drug resistance. Nat Commun.

[17] Gautschi O, Heighway J, Mack PC, Purnell PR, Lara PN, Gandara DR (2008). Aurora kinases as anticancer drug targets. Clin Cancer Res.

[18] Vader G, Lens SM (2008). The Aurora kinase family in cell division and cancer. Biochim Biophys Acta.

[19] Willems E, Dedobbeleer M, Digregorio M, Lombard A, Lumapat PN, Rogister B (2018). The functional diversity of Aurora kinases: a comprehensive review. Cell Div.

[20] Shah KN, Bhatt R, Rotow J, Rohrberg J, Olivas V, Wang VE (2019). Aurora kinase A drives the evolution of resistance to third-generation EGFR inhibitors in lung cancer. Nat Med.

[21] Levinson NM (2018). The multifaceted allosteric regulation of Aurora kinase A. Biochem J.

[22] Gavriilidis P, Giakoustidis A, Giakoustidis D (2015). Aurora kinases and potential medical applications of aurora kinase inhibitors: a review. J Clin Med Res.

[23] Bavetsias V, Linardopoulos S (2015). Aurora kinase inhibitors: current status and outlook. Front Oncol.

[24] Dar AA, Goff LW, Majid S, Berlin J, El-Rifai W (2010). Aurora kinase inhibitors-rising stars in cancer therapeutics?. Mol Cancer Ther.

[25] Dar AA, Belkhiri A, Ecsedy J, Zaika A, El-Rifai W (2008). Aurora kinase A inhibition leads to p73-dependent apoptosis in p53-deficient cancer cells. Cancer Res.

[26] Sehdev V, Peng D, Soutto M, Washington MK, Revetta F, Ecsedy J (2012). The aurora kinase A inhibitor MLN8237 enhances cisplatin-induced cell death in esophageal adenocarcinoma cells. Mol Cancer Ther.

[27] Falchook GS, Bastida CC, Kurzrock R (2015). Aurora kinase inhibitors in oncology clinical trials: current state of the progress. Semin Oncol.

[28] Nikhil K, Kamra M, Raza A, Haymour HS, Shah K (2020). Molecular interplay between AURKA and SPOP dictates CRPC pathogenesis via androgen receptor. Cancers.

[29] Wu J, Lee SW, Zhang X, Han F, Kwan SY, Yuan X (2013). Foxo3a transcription factor is a negative regulator of Skp2 and Skp2 SCF complex. Oncogene.

[30] Xu J, Zhou W, Yang F, Chen G, Li H, Zhao Y (2017). The beta-TrCP-FBXW2-SKP2 axis regulates lung cancer cell growth with FBXW2 acting as a tumour suppressor. Nat Commun.

[31] Li F, Dong X, Lin P, Jiang J (2018). Regulation of Akt/FoxO3a/Skp2 axis is critically involved in berberine-induced cell cycle arrest in hepatocellular carcinoma cells. Int J Mol Sci.

[32] Wang F, Chan CH, Chen K, Guan X, Lin HK, Tong Q (2012). Deacetylation of FOXO3 by SIRT1 or SIRT2 leads to Skp2-mediated FOXO3 ubiquitination and degradation. Oncogene.

[33] Goldberg SL, Fenaux P, Craig MD, Gyan E, Lister J, Kassis J (2014). An exploratory phase 2 study of investigational Aurora A kinase inhibitor alisertib (MLN8237) in acute myelogenous leukemia and myelodysplastic syndromes. Leuk Res Rep.

[34] Zhang W, Xia D, Li Z, Zhou T, Chen T, Wu Z (2019). Aurora-A/ERK1/2/mTOR axis promotes tumor progression in triple-negative breast cancer and dual-targeting Aurora-A/mTOR shows synthetic lethality. Cell Death Dis.

[35] Murugan AK (2019). mTOR: role in cancer, metastasis and drug resistance. Semin Cancer Biol.

[36] Xu J, Yue CF, Zhou WH, Qian YM, Zhang Y, Wang SW (2014). Aurora-A contributes to cisplatin resistance and lymphatic metastasis in non-small cell lung cancer and predicts poor prognosis. J Transl Med.

[37] Xu J, Wu X, Zhou WH, Liu AW, Wu JB, Deng JY (2013). Aurora-A identifies early recurrence and poor prognosis and promises a potential therapeutic target in triple negative breast cancer. PLoS ONE.

[38] Zhou W, Xu J, Li H, Xu M, Chen ZJ, Wei W (2017). Neddylation E2 UBE2F promotes the survival of lung cancer cells by activating CRL5 to degrade NOXA via the K11 linkage. Clin Cancer Res.

[39] Wan XB, Long ZJ, Yan M, Xu J, Xia LP, Liu L (2008). Inhibition of Aurora-A suppresses epithelial-mesenchymal transition and invasion by downregulating MAPK in nasopharyngeal carcinoma cells. Carcinogenesis.

[40] Zhang W, Xu J, Ji D, Li Z, He W, Yang F (2017). CyclinG1 amplification enhances aurora kinase inhibitor-induced polyploid resistance and inhibition of Bcl-2 pathway reverses the resistance. Cell Physiol Biochem.

[41] Zhou W, Xu J, Gelston E, Wu X, Zou Z, Wang B (2015). Inhibition of Bcl-xL overcomes polyploidy resistance and leads to apoptotic cell death in acute myeloid leukemia cells. Oncotarget.

