# Supplementary figures and images for: Aurora-A/FOXO3A/SKP2 axis promotes tumor progression in clear cell renal cell carcinoma and dual-targeting Aurora-A/SKP2 shows synthetic lethality
Source: Cell Death Dis. 2022 Jul 13;13(7):606. doi: 10.1038/s41419-022-04973-9 (PMC9279325; doi:10.1038/s41419-022-04973-9)

**Figure-1**

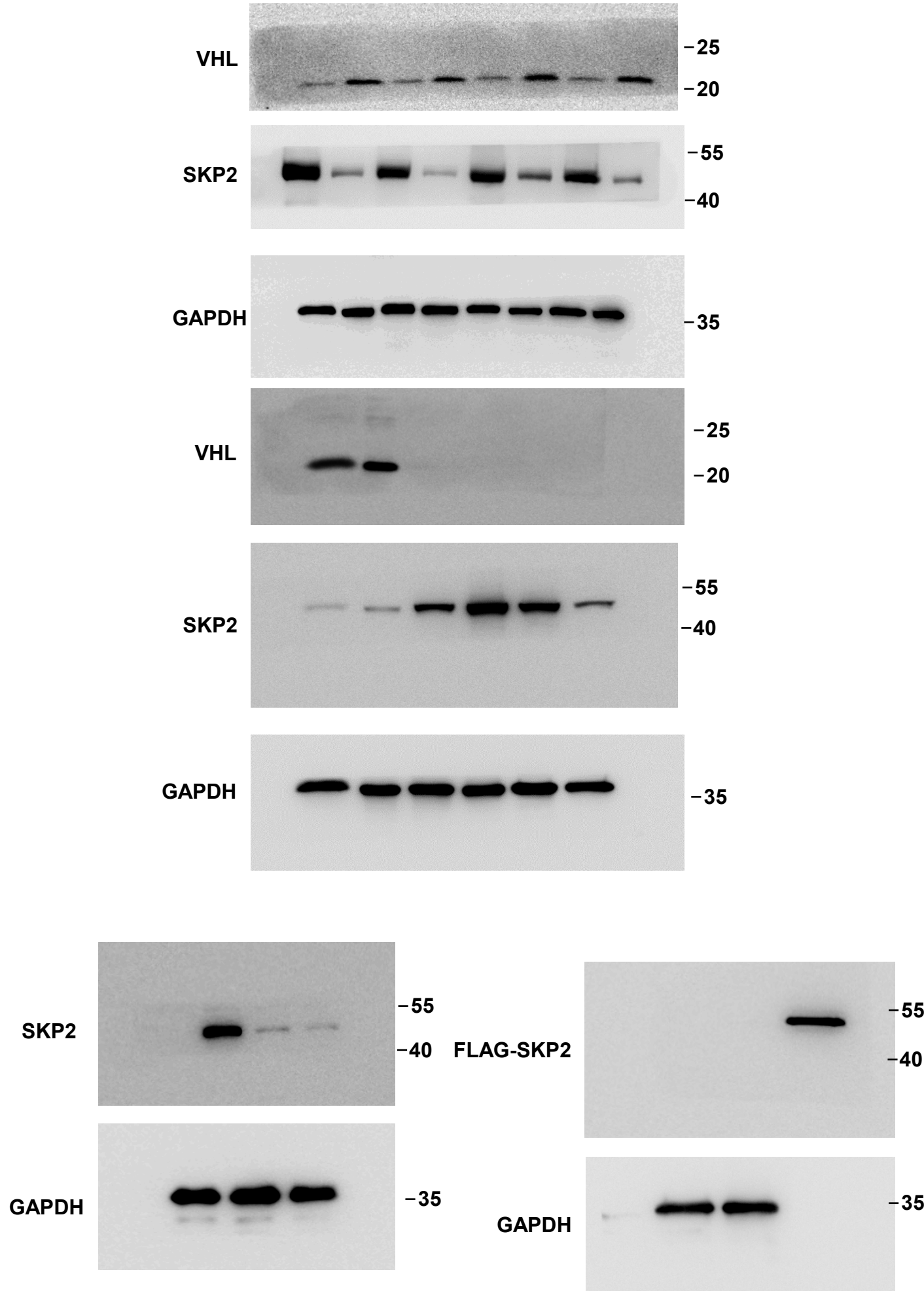

**Figure-2**

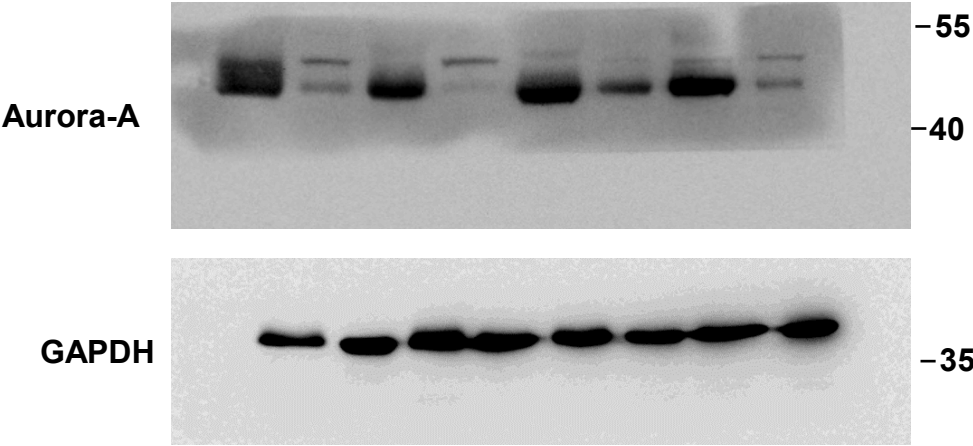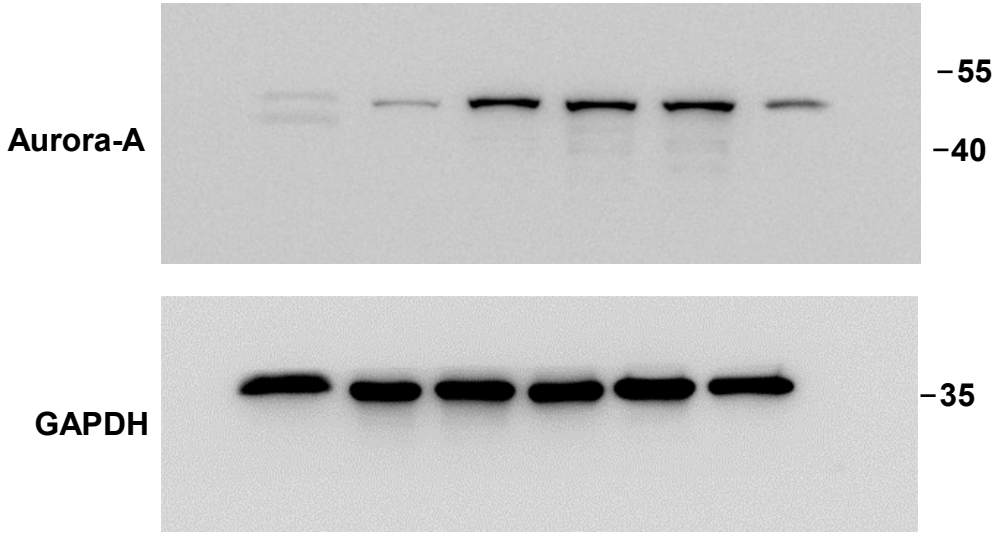

Figure-3

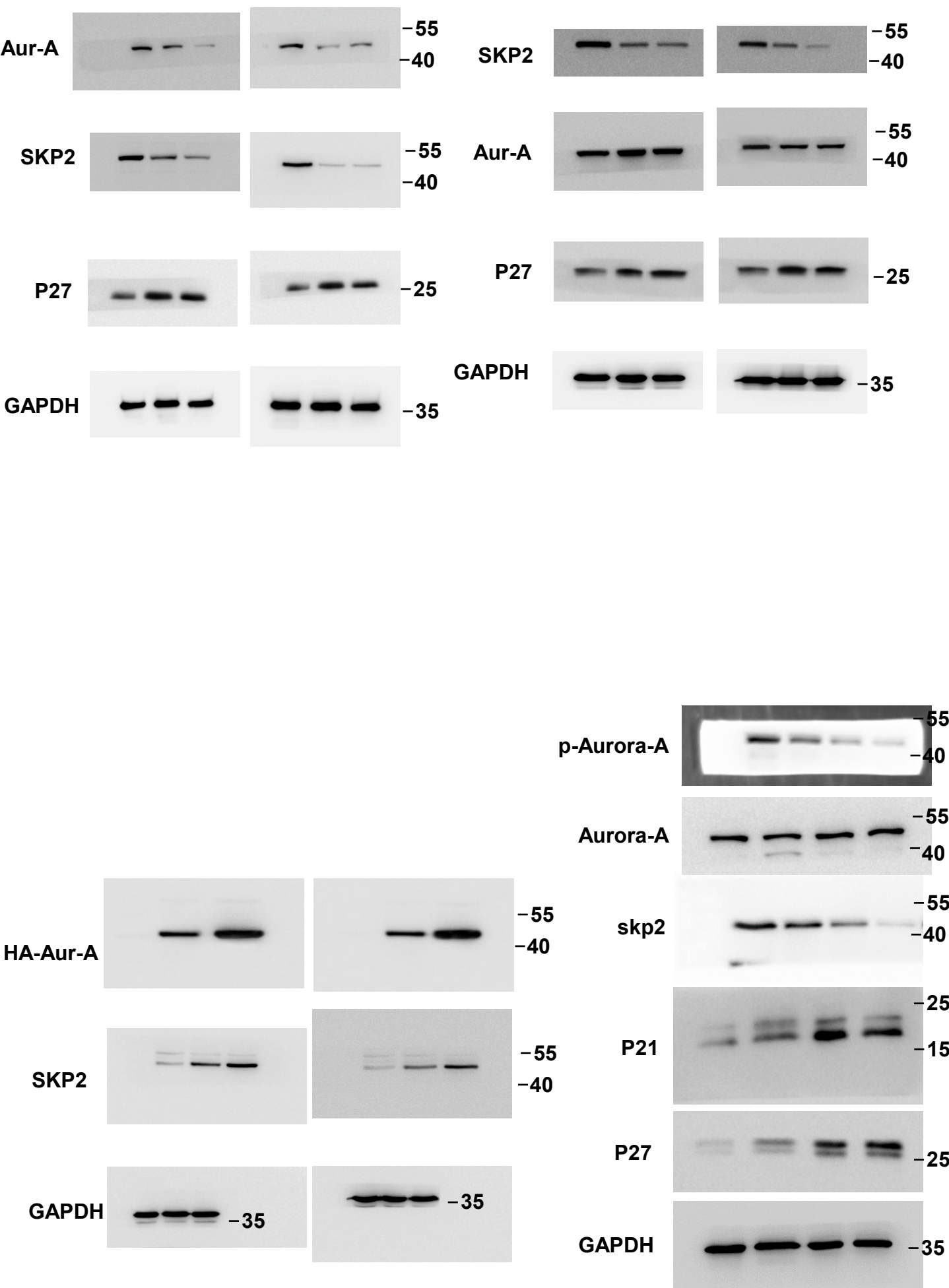

**Figure-4**

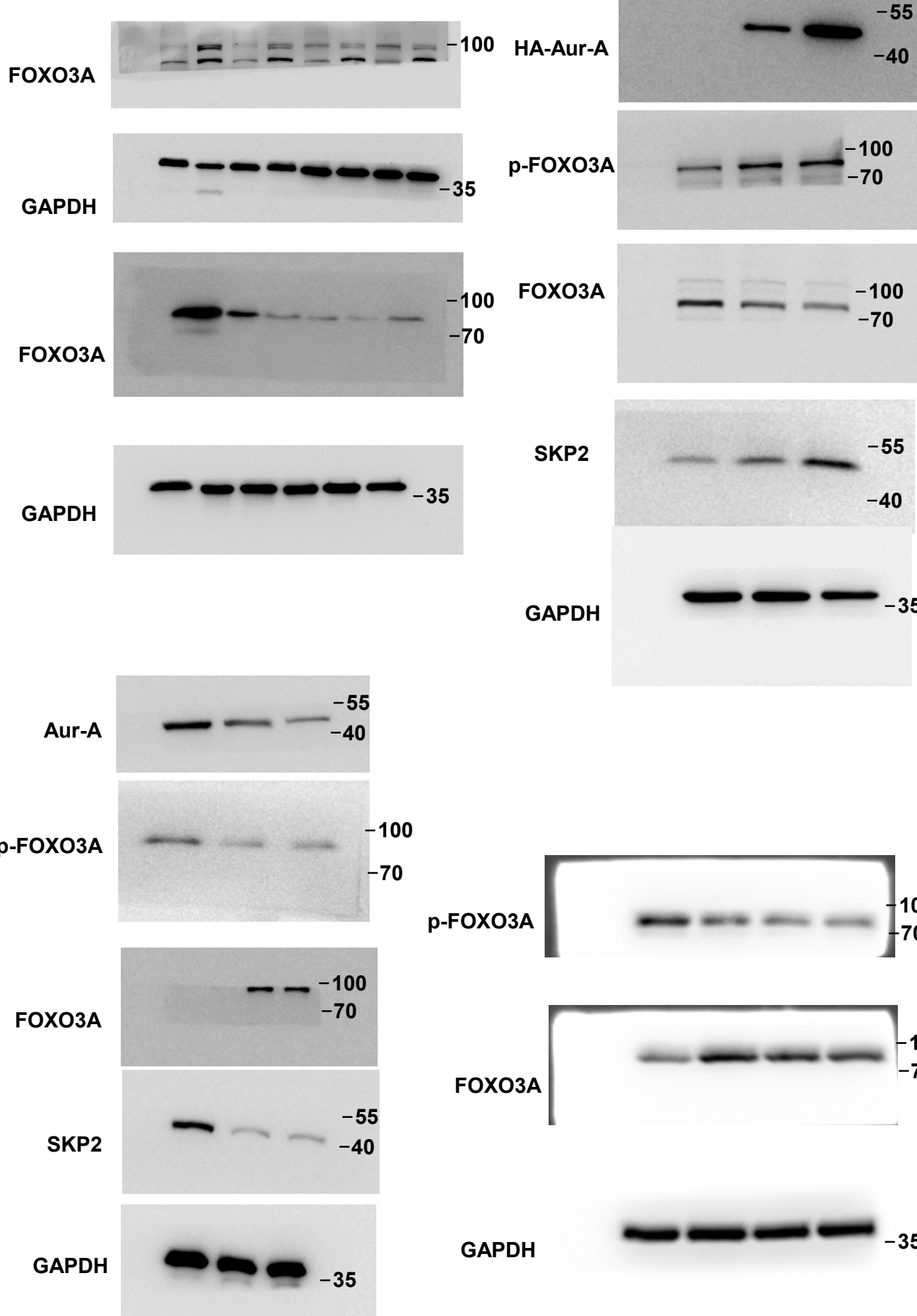

**Figure-5**

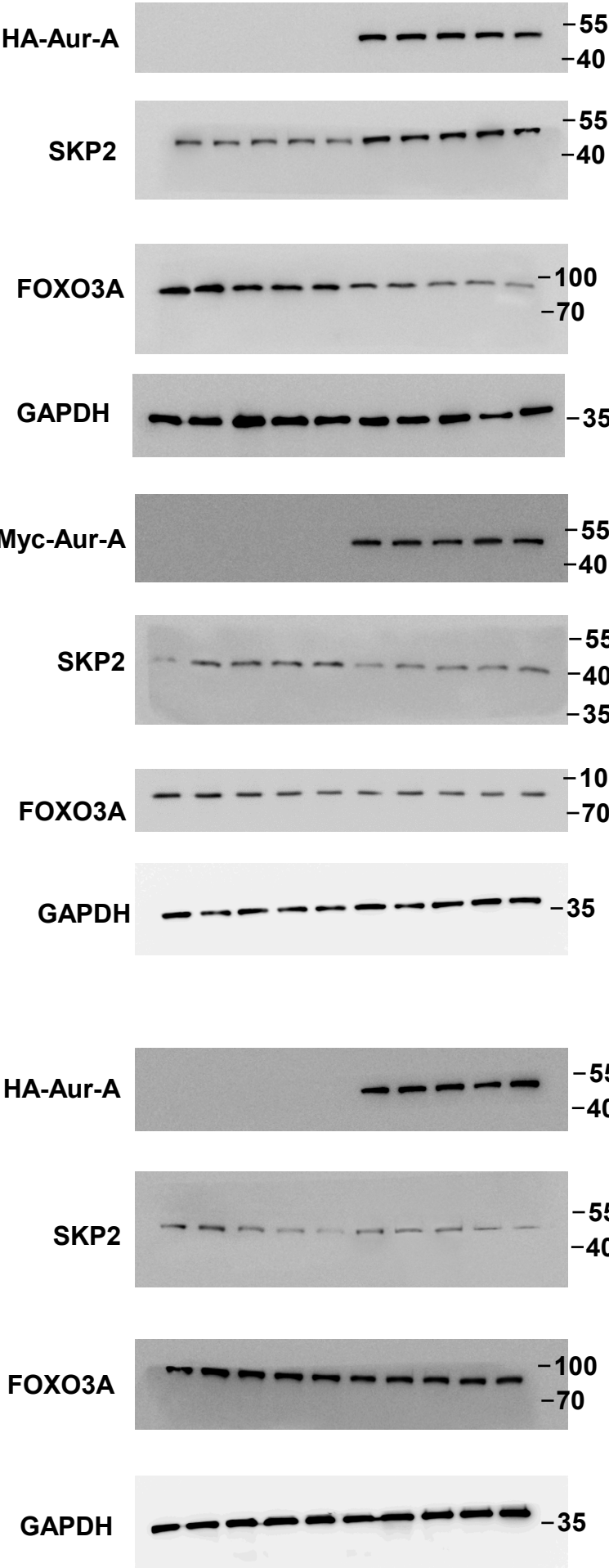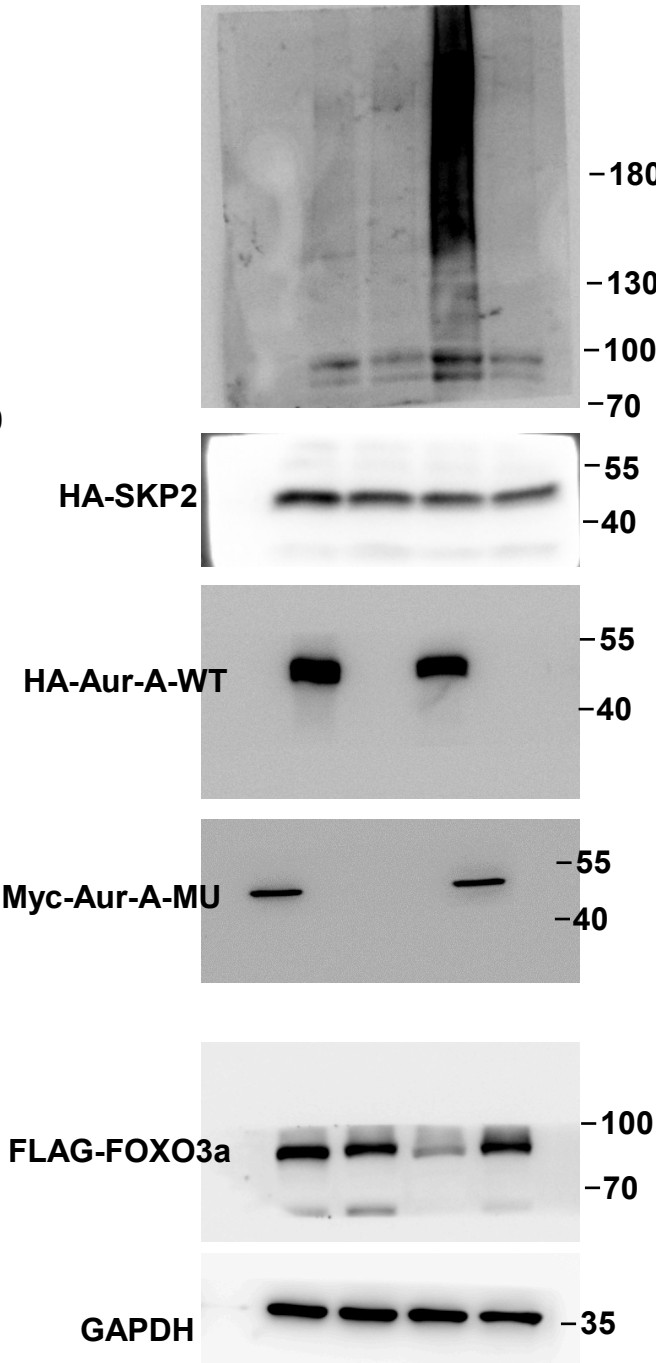

**Figure-6**

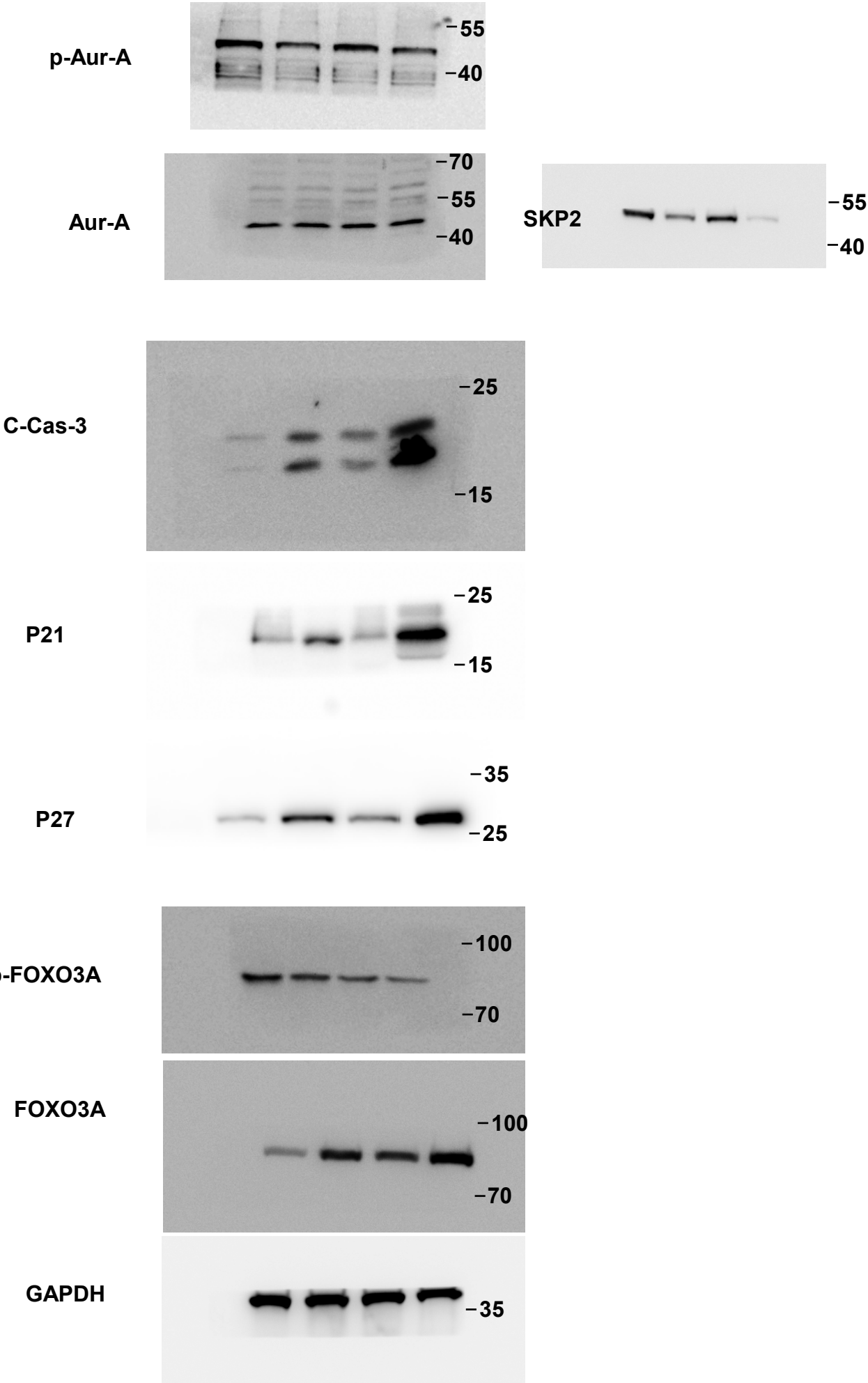

Supplement: Supplementary file 2 — Original Data File [file 41419_2022_4973_MOESM2_ESM.pdf]
